# Probabilistic pathway-based multimodal factor analysis

**DOI:** 10.1093/bioinformatics/btae216

**Published:** 2024-06-28

**Authors:** Alexander Immer, Stefan G Stark, Francis Jacob, Ximena Bonilla, Tinu Thomas, André Kahles, Sandra Goetze, Emanuela S Milani, Bernd Wollscheid, The Tumor Profiler Consortium, Rudolf Aebersold, Melike Ak, Faisal S Al-Quaddoomi, Silvana I Albert, Jonas Albinus, Ilaria Alborelli, Sonali Andani, Per-Olof Attinger, Marina Bacac, Daniel Baumhoer, Beatrice Beck-Schimmer, Niko Beerenwinkel, Christian Beisel, Lara Bernasconi, Anne Bertolini, Bernd Bodenmiller, Ximena Bonilla, Lars Bosshard, Byron Calgua, Ruben Casanova, Stéphane Chevrier, Natalia Chicherova, Ricardo Coelho, Maya D'Costa, Esther Danenberg, Natalie R Davidson, Monica-Andreea Drăgan, Reinhard Dummer, Stefanie Engler, Martin Erkens, Katja Eschbach, Cinzia Esposito, André Fedier, Pedro F Ferreira, Joanna Ficek-Pascual, Anja L Frei, Bruno Frey, Sandra Goetze, Linda Grob, Gabriele Gut, Detlef Günther, Pirmin Haeuptle, Viola Heinzelmann-Schwarz, Sylvia Herter, Rene Holtackers, Tamara Huesser, Alexander Immer, Anja Irmisch, Francis Jacob, Andrea Jacobs, Tim M Jaeger, Katharina Jahn, Alva R James, Philip M Jermann, André Kahles, Abdullah Kahraman, Viktor H Koelzer, Werner Kuebler, Jack Kuipers, Christian P Kunze, Christian Kurzeder, Kjong-Van Lehmann, Mitchell Levesque, Ulrike Lischetti, Flavio C Lombardo, Sebastian Lugert, Gerd Maass, Markus G Manz, Philipp Markolin, Martin Mehnert, Julien Mena, Julian M Metzler, Nicola Miglino, Emanuela S Milani, Holger Moch, Simone Muenst, Riccardo Murri, Charlotte K Y Ng, Stefan Nicolet, Marta Nowak, Monica Nunez Lopez, Patrick G A Pedrioli, Lucas Pelkmans, Salvatore Piscuoglio, Michael Prummer, Prélot Laurie, Natalie Rimmer, Mathilde Ritter, Christian Rommel, María L Rosano-González, Gunnar Rätsch, Natascha Santacroce, Jacobo Sarabia del Castillo, Ramona Schlenker, Petra C Schwalie, Severin Schwan, Tobias Schär, Gabriela Senti, Wenguang Shao, Franziska Singer, Sujana Sivapatham, Berend Snijder, Bettina Sobottka, Vipin T Sreedharan, Stefan Stark, Daniel J Stekhoven, Tanmay Tanna, Alexandre P A Theocharides, Tinu M Thomas, Markus Tolnay, Vinko Tosevski, Nora C Toussaint, Mustafa A Tuncel, Marina Tusup, Audrey Van Drogen, Marcus Vetter, Tatjana Vlajnic, Sandra Weber, Walter P Weber, Rebekka Wegmann, Michael Weller, Fabian Wendt, Norbert Wey, Andreas Wicki, Mattheus H E Wildschut, Bernd Wollscheid, Shuqing Yu, Johanna Ziegler, Marc Zimmermann, Martin Zoche, Gregor Zuend, Gunnar Rätsch, Kjong-Van Lehmann

**Affiliations:** Biomedical Informatics Group, Department of Computer Science, ETH Zurich, 8092 Zurich, Switzerland; Max Planck Institute for Intelligent Systems, 72076 Tübingen, Germany; Biomedical Informatics Group, Department of Computer Science, ETH Zurich, 8092 Zurich, Switzerland; Swiss Institute of Bioinformatics, 1015 Lausanne, Switzerland; Ovarian Cancer Research, Department of Biomedicine, University Hospital Basel and University of Basel, 4031 Basel, Switzerland; Biomedical Informatics Group, Department of Computer Science, ETH Zurich, 8092 Zurich, Switzerland; Swiss Institute of Bioinformatics, 1015 Lausanne, Switzerland; Biomedical Informatics Group, Department of Computer Science, ETH Zurich, 8092 Zurich, Switzerland; Swiss Institute of Bioinformatics, 1015 Lausanne, Switzerland; Biomedical Informatics Group, Department of Computer Science, ETH Zurich, 8092 Zurich, Switzerland; Swiss Institute of Bioinformatics, 1015 Lausanne, Switzerland; Swiss Institute of Bioinformatics, 1015 Lausanne, Switzerland; Institute of Translational Medicine, Department of Health Sciences and Technology, ETH Zurich, 8093 Zurich, Switzerland; ETH PHRT Swiss Multi-Omics Center (SMOC), 8093 Zurich, Switzerland; Swiss Institute of Bioinformatics, 1015 Lausanne, Switzerland; Institute of Translational Medicine, Department of Health Sciences and Technology, ETH Zurich, 8093 Zurich, Switzerland; Swiss Institute of Bioinformatics, 1015 Lausanne, Switzerland; Institute of Translational Medicine, Department of Health Sciences and Technology, ETH Zurich, 8093 Zurich, Switzerland; Biomedical Informatics Group, Department of Computer Science, ETH Zurich, 8092 Zurich, Switzerland; Swiss Institute of Bioinformatics, 1015 Lausanne, Switzerland; AI Center at ETH Zurich, 8092 Zurich, Switzerland; Biomedical Informatics Research, University Hospital Zurich, 8006 Zurich, Switzerland; Department of Biology, ETH Zurich, 8049 Zurich, Switzerland; Biomedical Informatics Group, Department of Computer Science, ETH Zurich, 8092 Zurich, Switzerland; Cancer Research Center Cologne Essen, University Hospital Cologne, 50937 Cologne, Germany; Joint Research Center for Computational Biomedicine, University Hospital RWTH Aachen, 52074 Aachen, Germany

## Abstract

**Motivation:**

Multimodal profiling strategies promise to produce more informative insights into biomedical cohorts via the integration of the information each modality contributes. To perform this integration, however, the development of novel analytical strategies is needed. Multimodal profiling strategies often come at the expense of lower sample numbers, which can challenge methods to uncover shared signals across a cohort. Thus, factor analysis approaches are commonly used for the analysis of high-dimensional data in molecular biology, however, they typically do not yield representations that are directly interpretable, whereas many research questions often center around the analysis of pathways associated with specific observations.

**Results:**

We develop PathFA, a novel approach for multimodal factor analysis over the space of pathways. PathFA produces integrative and interpretable views across multimodal profiling technologies, which allow for the derivation of concrete hypotheses. PathFA combines a pathway-learning approach with integrative multimodal capability under a Bayesian procedure that is efficient, hyper-parameter free, and able to automatically infer observation noise from the data. We demonstrate strong performance on small sample sizes within our simulation framework and on matched proteomics and transcriptomics profiles from real tumor samples taken from the Swiss Tumor Profiler consortium. On a subcohort of melanoma patients, PathFA recovers pathway activity that has been independently associated with poor outcome. We further demonstrate the ability of this approach to identify pathways associated with the presence of specific cell-types as well as tumor heterogeneity. Our results show that we capture known biology, making it well suited for analyzing multimodal sample cohorts.

**Availability and implementation:**

The tool is implemented in python and available at https://github.com/ratschlab/path-fa

## 1 Introduction

A current trend in biomedical research is to obtain multiple types of molecular data for a given sample (Boehm *et al.* 2022). These modalities represent different views of the molecular landscape, each measuring different aspects, resolutions, and scales to provide us with complementary information that helps us understand the relationships between molecular mechanisms. A common question regarding multimodal data is to identify factors that explain the range of observed measurements (e.g. gene or protein expression, copy number variations, phosphorylation, etc.) across a given sample population (Ritchie *et al.* 2015). In molecular oncology, related approaches are used to quantify tumor composition and immune cell content from bulk measurement technologies, leveraging shared signals observed across a larger cohort (Chen *et al.* 2018).

Thus, extracting useful representations of samples from omics data that are biologically relevant, correlate with cell-type abundance, or even clinical variables, remains an important challenge. PLIER (Mao *et al.* 2019) and MultiPLIER (Taroni *et al.* 2019) leverage pathway level information to analyze factors that drive the differences in biology observed within a cohort that significantly improve interpretability compared to gene level approaches, but only apply to a single modality of transcriptomics data. MOFA (Argelaguet *et al.* 2018) conversely, is a multimodal factor analysis approach but operates on a potentially unidentifiable latent space instead of pathways, which can make interpretation challenging. By making less assumptions about the latent factors, it also requires more samples to infer them.

Here, we propose a novel approach for a multimodal factor analysis that operates on the level of biological pathways to integrate the information from multiple modalities. We leverage the concepts from PLIER and MOFA into a novel single framework, PathFA. We use pathway information to integrate multiple modalities into the same factor analysis model. This new multi-omics factor analysis is able to join *proteomics and transcriptomics* (RNA) data in the space of pathways (PLIER is designed for RNA) instead of unknown latent factors (MOFA). To effectively integrate and balance different observed markers and modalities, we propose a probabilistic pathway-based factor analysis model and efficient Bayesian inference method.

Using PathFA, we show that both (1) the addition of another data modality and (2) utilization of prior knowledge improve reconstruction in a simulation and downstream performance on real data, for example correlation with cell types. Specifically, we improve over MOFA when it is possible to incorporate prior knowledge in form of pathways and over PLIER when multiple omics are available. On matched proteomics and transcriptomics data of the Tumor Profiler Consortium (Irmisch *et al.* 2021; The Tumor Profiler Consortium 2024a, 2024b), the latent factors of PathFA align with relevant pathways for clinical outcome, tumor heterogeneity, and cell-type composition. Further, we find that proteomics data by itself can be as useful as transcriptomics in our case, although pathways are originally derived on RNA markers.

In summary, in this study, we make the following main contributions:

1. We introduce PathFA, a novel probabilistic factor analysis model integrating multimodal data (transcriptomics and proteomics) using biological pathways.
2. We implemented an efficient Bayesian inference method for automatic hyper-parameter optimization, enhancing the analysis of high-dimensional molecular data.
3. We demonstrate improved interpretability of molecular datasets by anchoring analysis in known biological pathways.
4. We validate PathFA with real-world patient data from the Tumor Profiler consortium, successfully identifying key biological insights related to cell-type composition, survival, and tumor heterogeneity.

## 2 Methods

### 2.1 Data and preprocessing

We use the transcriptomics (RNA-Seq) and proteomics data (DIA-MS) data (Xuan *et al.* 2020) generated from Melanoma (The Tumor Profiler Consortium 2024b) and Ovarian patients (The Tumor Profiler Consortium 2024a), as part of the Tumor Profiler(TuPro) study (Irmisch *et al.* 2021). TuPro is a multicenter study that has deeply phenotyped metastatic tumor across multiple indications (Irmisch *et al.* 2021). All data will be made available upon release of the Tumor Profiler Marker papers.

#### 2.1.1 Sample selection for transcriptomics and proteomics

Based on the transcriptomics and proteomics data from tumor profiler, we selected a subset of samples for ovarian cancer and melanoma. RNA-seq library preparation was done using the Illumina TruSeq Standed Total RNA Sample Preparation kit (Ribo-Zero Gold). Nova-Seq 6000 was used to sequence the samples. Proteomics data was generated via a Data Independent Acquisition Mass Spectrometry approach. We selected a subcohort of patients that had passed the internal quality control of both transcriptomics and proteomics nodes. RNA-seq data was filtered based on RIN score and fastqc metrics. Samples failed QC when the RIN score was below six, or if three or more FASTQC modules were failed. For proteomics data, quality control was assessed in context of reference control samples [mix of three Ovarian cell lines (Kuramochi, OVCA3, SKOV3) or for melanoma (MKFY6-1-P15/MKFY6-1-P15/MTG5K-1-P19)]. Samples with more than 70%, as well as peptides with more than 75%, missing values were removed. We also filtered for samples that had both modalities profiled, and a complete set of the relevant metadata. This left us with 42 ovarian samples and 34 melanoma samples.

#### 2.1.2 Data preprocessing

Both transcriptomics and proteomics data are processed similarly and according to standard practice. In both cases, the raw data are first standardized by the library size, i.e. the total number of counts per sample. For RNA, this is equivalent to reads per million mapped reads (RPM). The counts are transformed with the following function: log(1 + x), then, quantile normalized, and *z*-scored. The quantile normalization ensures that the transformed counts for each sample roughly follow a normal distribution and *z*-scoring standardizes per marker. The preprocessing is identical to Mao *et al.* (2019) with the difference that we use log(1 + x) instead of filtering genes with low counts and applying log(x). The melanoma dataset had 19 612 genes and 4651 proteins. The ovarian dataset had 19 965 genes and 4068 proteins.

The prior knowledge about pathways is extracted from the Molecular Signatures Database (MSigDB; Liberzon *et al.* 2011) in addition to a *curated* set of pathways, developed by the Tumor Profiler Consortium for the analysis of melanoma pathways (Irmisch *et al.* 2021). From MSigDB, we use Hallmark (Liberzon *et al.* 2015) and cell-type signature genesets. However, these are specified in terms of contained genes only. To use the same pathways for proteomics data, we translate these pathways into the corresponding markers, mapping to Emsembl gene ids (Ruffier *et al.* 2017) via UniProt (Consortium 2019).

#### 2.1.3 Synthetic data-generating process

**Figure 1.**
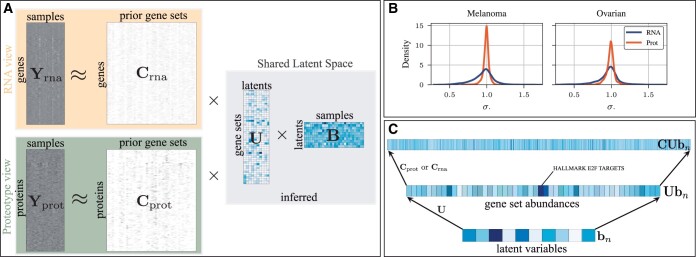
Schematic overview of our method. (A) Samples are hierarchically represented via pathways and latents inferred jointly from transcriptomics (RNA) and proteomics observations. Corresponding prior pathways translate into the space of both observed modalities. (B) Density plot of observation noises for both RNA and proteomics data shows heteroscedasticity within and across modalities. Proteomics markers have less varying precision while RNA has more spread. (C) Hierarchy of representations for a single sample. A sample is represented by a low-dimensional latent variable, projected into pathway abundances, which can be reconstructed into both proteomics and RNA observations.

To generate synthetic data for simulation experiments, we use MSigDB Hallmark pathways, which provide us with 50 pathways and corresponding transcriptomics and translated (Section 2.1.2) proteomics markers. Further, each pathway is associated to one of eight process categories, e.g. development, DNA damage, or immune, which we use as ground truth latent variables. This allows us to generate data by simulating loadings from a mixture of three isotropic Gaussians, simulating clusters of samples. The mean of each cluster is itself sampled from an isotropic Gaussian with higher variance. Further, we use a heteroscedastic observation noise on the markers that follows the shape of observed data in Tumor Profiler data. The average transcriptomics observation noise is 0.95 and that of Proteomics is 0.98 while the spread of noises on the marker level is smaller for Proteomics as shown in Fig. 1B.

### 2.2 Probabilistic pathway-based factor analysis

PathFA uses prior information in terms of associations of pathways to either gene or protein markers. This allows our model to use the pathways as a latent space based on prior knowledge. PLIER (Mao *et al.* 2019) uses the same prior information in their model but only for transcriptomics data and in combination with a standard factor analysis (FA) model. Below, we introduce our method that is both applicable to transcriptomics and proteomics data and we extend it to the multimodal setting (observing both per sample) in Section 2.4.

We denote the data of *m* gene or protein markers for *n* samples as matrix $Y\in R^{m\;\times \;n}$. Further, we assume prior knowledge about *p* relevant pathways that each consist of several markers (gene or protein) as $C\in {\left\{0,1\right\}}^{m\;\times \;p}$ with $C_{m,p}=1$ if the *m*th marker is part of the *p*th pathway and otherwise $C_{mp}=0$. PathFA infers two parameter matrices: $U\in R_{\geq 0}^{p\;\times \;k}$ associates the pathways to latent variables such that $U_{p,k}$ denotes the relevance of the *p*th pathway to the *k*th latent variable. Associating latent variables through pathways allows to interpret them better than in a standard factor analysis. Finally, each column of the matrix $B\in R^{k\;\times \;n}$ specifies the *k*-dimensional loadings per sample. Informally, we model the observations Y with CUB. Typically, we have *m* markers in the order of thousands, *p* pathways in the order of hundreds, and tens of *k* latent dimensions.

PathFA uses a Gaussian likelihood function with learnable observation noises per marker. This allows PathFA to ignore noisy markers and focus on the informative ones as they are actually heteroscedastic (Argelaguet *et al.* 2018). The observation noise is parameterized by $\sigma \in R_{+}^{m}$ with $\sigma {\;}_{m}$ denoting the observation noise of the *m*th marker. The likelihood function is then given by

(1) $$p(Y|U,B,\sigma )=\prod_{m,n}\;N(Y_{m,n}|c_{m}^{T}Ub_{n},\sigma {\;}_{m}^{2}),$$

where $c_{m}$ is the vector denoting the *m*th row of C and $b_{n}$ the *n*th column of B. The likelihood governs that each marker of a sample is reconstructed by the pathways it belongs to ($c_{m}$) summed over the samples’ abundance of the respective pathways ($Ub_{n}$). This reconstruction is visualized in Fig. 1C. In comparison to standard factor analysis, all observations are reconstructed through the pathways. We additionally still maintain lower-dimensional latent variables in B corresponding to the *loadings* in factor analysis.

We place independent zero-mean Gaussian priors over the parameter matrices to ensure sparsity of the pathway-latent association U and to identify the relevant latent dimensions in B. For U, we use the identically-sized matrix of prior precisions $\Lambda \in R_{+}^{p\;\times \;k}$ with entries $\Lambda_{p,k}$ denoting the regularization strength of the entry $U_{p,k}$ akin to automatic relevance determination (ARD). ARD is used commonly for biological latent-variable models to achieve sparsity in a probabilistic framework (Li *et al.* 2002; Tan and Févotte 2012). Its advantage is that we do not have to set a sparsity level or regularization hyperparameter *a priori* but can infer it automatically. The precision of B is controlled per latent variable with vector $\delta \in R_{+}^{k}$. Mathematically, we have the priors

(2) $$p(U|\Lambda )=\prod_{p,k}\;N(U_{p,k};0,\Lambda_{p,k}^{-1}),\;\;p(B|\delta )=\prod_{k,n}\;N(B_{k,n};0,\delta_{k}^{-1}).$$

To infer U and B, we find their *maximum a-posteriori* (MAP) values by minimizing their negative log joint distribution with the observations,

(3) $$\begin{array}{l} L(U,B)=-\text{log}\;p(Y,U,B|\sigma ,\Lambda ,\delta )\\ \begin{array}{c} =-\text{log}\;p(Y|U,B,\sigma )-\text{log}\;p(U|\Lambda )-\text{log}\;p(B|\delta ). \end{array} \end{array}$$

To optimize this objective, we use alternating least-squares (ALS) (Hastie *et al.* 2015), which uses alternating closed-form updates for the parameters. The inference procedure is detailed in Section 2.3.

To select the hyperparameters of the model that control the observation noises σ, and relevance parameters Λ,δ, we use a Bayesian model selection scheme, which, perhaps surprisingly, does not increase the complexity of our algorithm. For example with cross-validation, choosing relevance parameters would be intractable due to the curse of dimensionality as we have m + pk + k hyperparameters. In our experiments, this can be more than $10^{4}$. For our hyperparameter optimization, we use a Laplace approximation to the marginal likelihood that has been successfully used in automatic relevance determination (MacKay 1994) and can jointly optimize observation noises σ and relevance parameters Λ,δ. In particular, we maximize the evidence of the hyperparameters,

(4) $$\begin{array}{c} \;\text{log}\;p(Y|\sigma ,\Lambda ,\delta )\approx \;\text{log}\;p(Y,U_{*},B_{*}|\sigma ,\Lambda ,\delta )\\ \begin{array}{c} -\frac{1}{2}\text{log}\;\left|\frac{1}{2\pi }\nabla_{U,B}^{2}L(U_{*},B_{*})\right|, \end{array} \end{array}$$

where the Hessian is evaluated at MAP estimates $U_{*}$ and $B_{*}$. We further approximate the Hessian as block-diagonal individually for U and B, which typically performs as well but has significant computational benefits (Immer *et al.* 2021). The hyperparameters are updated in a closed-form procedure similar to MacKay (1994) and Tipping (2001). Interestingly, the closed-form updates require computation of the same quantities as required for the alternating least-squares updates and thus do not increase overall computational complexity. That is, ARD is for free in our model when we use ALS updates for the parameters.

Algorithm 1. Probabilistic Multi-Path-FA

**Require:** observations $Y_{r}\in R^{m_{r}\;\times \;n},Y_{p}\in R^{m_{p}\;\times \;n}$, pathway knowledge $C_{r}\in {\left\{0,1\right\}}^{m_{r}\;\times \;p},C_{p}\in {\left\{0,1\right\}}^{m_{p}\;\times \;p}$, N > 0 iterations, γ > 0 step size, k > 0 number of latents

1: $U\leftarrow 0\in R_{+}^{p\;\times \;k};B\leftarrow \mathrm{truncSVD}(Y_{r},k)$  ▹ init. params

2: $r\leftarrow \mathrm{logspace}(-4,4,k);c_{r}\leftarrow 1;c_{p}\leftarrow 1$

3: $\delta \leftarrow r\in R_{+}^{k}$; $\Lambda \leftarrow 1_{p}⊗r\in R_{+}^{p\;\times \;k}$  ▹ init. prior precision

4: $\sigma {\;}_{r}\leftarrow 1\in R_{+}^{m_{r}}$; $\sigma {\;}_{p}\leftarrow 1\in R_{+}^{m_{p}}$  ▹ init. obs. noise

5: **for**  n∈[1,…,N]  **do**

6:   **if** *n* is even **then**  ▹ Alternating least-squares

7:    Compute gradient and Hessian $G_{m}(B),H_{m}(B)$

8:    $B\leftarrow B-\gamma H_{m}{\left(B\right)}^{-1}\mathrm{vec}(G_{m}(B))$

9:   **else**

10:    Compute gradient and Hessian $G_{m}(U),H_{m}(U)$

11:    $U\leftarrow U-\gamma H_{m}{\left(U\right)}^{-1}\mathrm{vec}(G_{m}(U))$

12:    Project U to non-negative by clamping to zero

13: Update $\Lambda ,\delta ,\sigma {\;}_{r},\sigma {\;}_{p}$ as in Equations (9) and (10) using $H_{m}(U),H_{m}(B)$

14: Closed-form update for scalar factors $c_{r},c_{p}$

### 2.3 Efficient inference of path-FA

We optimize the parameters of the model using *alternating least-squares* (ALS), which guarantees improvement in each step of the algorithm due to closed-form updates. Since both ALS and the Laplace approximation rely on the Hessian, computation is shared making hyperparameter optimization asymptotically free. Our updates resemble a Bayesian ALS that jointly optimizes parameters and hyperparameters by repeated integration as done for neural networks (Immer *et al.* 2021), and might be relevant outside the context of fitting PathFA, for example, in general matrix factorization or factor analysis problems. We first derive the gradients and Hessians of the parameter objective in Equation (3), which then allow to derive the parameter and hyperparameter updates.

#### 2.3.1 Gradients and Hessians

Defining $C_{\sigma \;}\overset{\text{def}}{=}\sigma {\;}^{-2}\;○\;C$, where ○ is the pointwise hadamard product, the gradients of the objective are given by

(5) $$\begin{array}{l} G(U)\overset{\text{def}}{=}\nabla_{U}L(U,B)=-C_{\sigma \;}^{T}(Y-CUB)B\;+\;\Lambda \;○\;U,\\ G(B)\overset{\text{def}}{=}\nabla_{B}L(U,B)=-U^{T}C_{\sigma \;}^{T}(Y-CUB)\;+\;(\delta ⊗1_{n})\;○\;B, \end{array}$$

where ⊗ denotes the Kronecker product and $1_{n}$ the *n*-dimensional vector of 1 s. For alternating least-squares and the marginal likelihood approximation, we further need the Hessians

(6) $$\begin{array}{c} H(U)\overset{\text{def}}{=}\nabla_{\mathrm{vec}(U)}^{2}L(U,B)=(C_{\sigma \;}^{T}C)⊗(BB^{T})\;+\;\text{diag}(\mathrm{vec}(\Lambda )),\\ H(B)\overset{\text{def}}{=}\nabla_{\mathrm{vec}(B)}^{2}L(U,B)=(U^{T}C_{\sigma \;}^{T}CU)⊗I_{n}\;+\;\text{diag}(\delta )⊗I_{n}\\ \;=(U^{T}C_{\sigma \;}^{T}CU\;+\;\text{diag}(\delta ))⊗I_{n}, \end{array}$$

where vec(Λ) concatenates the columns of Λ into a vector and diag(·) turns a vector into a diagonal matrix with elements given by the argument. The Kronecker-factored structure of the Hessian w.r.t. B allows for efficient computation of inverse and log-determinant required for ALS and marginal likelihood, respectively, by computing these quantities on the individual factors. However, the Hessian w.r.t. U requires evaluation of the Kronecker product for inversion due to the ARD prior controlled by Λ. This is not a problem because U’s shape is the number of pathways times number of latents, and therefore, small, i.e. below $10^{3}$.

#### 2.3.2 Updates and algorithm

We optimize the model parameters U and B efficiently using ALS, or equivalently Newton’s method, by preconditioning the gradient with the inverse of the Hessian. The hyperparameters are updated using the same quantities, therefore incurring no additional cost, and use fixed-point updates proposed by MacKay (1992) for neural network regression but we apply these updates already during training, which requires less time to converge (Immer *et al.* 2021). U is initialized to a zero-matrix and constrained to be non-negative and B is initialized using the right factor of a *k*-truncated singular-value decomposition of the observation matrix. The observation noises are initialized to a vector of 1 s and prior precisions logarithmically spaced between $10^{-2}$ and $10^{2}$ along the *k* latent variables.

The parameter updates are given by a Newton update with step size γ:

(7) $$M_{t\;+\;1}\leftarrow M_{t}-\gamma H{\left(M_{t}\right)}^{-1}\mathrm{vec}(G(M_{t})),$$

where M is either parameter U or B. For B this further simplifies due to the Kronecker-factored structure of its Hessian:

(8) $$B_{t\;+\;1}\leftarrow B_{t}-\gamma {\left(U_{t}^{T}C_{\sigma \;}^{T}CU_{t}\;+\;\text{diag}(\delta )\right)}^{-1}G(B),$$

which is efficient to compute since the left-multiplied Kronecker factor of the Hessian is quadratic in latents (k × k) and cheap to invert. To update U, we need to invert a matrix of size pk × pk, which is typically tractable and fast with $pk\approx 10^{3}$.

The hyperparameter updates rely on the inverse of the Hessian as well and therefore require no overhead computational complexity. The updates are similar to the ones for sparse Bayesian learning (MacKay 1992; Tipping 2001) but applied to our particular case of matrix factorization, which has considerably different priors and likelihoods. In particular, we have for the prior precisions Λ:

(9) $$\Lambda_{p,k}=\frac{1-\Lambda_{p,k}H{\left(U\right)}_{pk,pk}^{-1}}{U_{p,k}^{2}},$$

which requires the diagonal elements of the inverse of Us Hessian like the ALS update. For the prior precisions of B and observation noises, we have:

(10) $$\begin{array}{c} \delta_{k}=\frac{n(1-\delta_{k}{\left(U^{T}C_{\sigma \;}^{T}CU\;+\;\text{diag}(\delta )\right)}_{k,k}^{-1})}{\sum_{n}B_{k,n}^{2}},\\ \begin{array}{cc} \sigma {\;}_{m}^{2} & =\frac{\sum_{n}{\left(c_{m}Ub_{n}-Y_{m,n}\right)}^{2}}{n-p\;+\;\mathrm{tr}(\delta H^{-1})}. \end{array} \end{array}$$

Computing these updates poses no overhead in computation over the ALS updates and enables a hyperparameter-free algorithm. This is because only ALS requires a step size γ, which can be simply set to a common default of 0.1, and the hyperparameter updates are entirely in closed-form.

### 2.4 Multi-omics PathFA

To extend PathFA to simultaneous proteomics and transcriptomics data per sample, we only extend the likelihood but keep the same latent variables U and B, which construct a shared hierarchical latent space (see Fig. 1). Specifically, we have another view and thus likelihood for the proteomics data with translated pathway prior knowledge $C_{p}$. In principle, analyzing both modalities concurrently should enhance the precision of latent variable inference, even with a reduced number of samples. Moreover, the complementary nature of these modalities can potentially lead to more robust and effective latent representations, which may be beneficial for downstream tasks.

#### 2.4.1 Probabilistic model of multi-omics PathFA

Mathematically, we have prior knowledge about the gene-set-to-marker relationships in form of $C_{p}\in {\left\{0,1\right\}}^{m_{p}\;\times \;p}$ for $m_{p}$ protein markers and $C_{r}\in {\left\{0,1\right\}}^{m_{r}\;\times \;p}$ for $m_{r}$ RNA markers, respectively. Importantly, both cover the same pathways, which function as representation space. With proteomics observations $Y_{p}\in R^{m_{p}\;\times \;n}$ and transcriptomics observations $Y_{r}\in R^{m_{r}\;\times \;n}$, we have the likelihood

(11) $$p(Y_{p},Y_{r}|U,B,\sigma {\;}_{p},\sigma {\;}_{r})=p(Y_{p}|U,B,\sigma {\;}_{p})\;\times \;p(Y_{r}|U,B,\sigma {\;}_{r}),$$

which is simply the product of two unimodal likelihoods, which are defined in Equation (1). This means, we potentially observe more evidence for latent variables U and B. We model both observations as conditionally independent and with their own per-marker noise levels denoted by $\sigma {\;}_{r}$ and $\sigma {\;}_{p}$. Inferring noise levels is important to correctly weight the signal and noise coming from either modality. Further, modality-specific scalar factors $c_{r}$ and $c_{p}$ are multiplied to reconstruct the respective modalities, which can absorb potential inconsistencies in preprocessing or normalization of transcriptomics and proteomics data, respectively. Both scalars can be updated in a closed form and have no prior. The Gaussian priors with zero mean and precision parameters $\lambda_{U},\lambda_{B}$ on U and B are as in Equation (2).

#### 2.4.2 Inference for multi-omics PathFA

The alternating least-squares parameter updates for multimodal PathFA and the corresponding hyperparameter updates are only slightly different from the ones of the unimodal variant described above. In particular, the additional observation in the multimodal likelihood [Equation (11)] gives an additional summand in the gradient and Hessian computation, which are given in Equations (5) and (6) for the unimodal case, respectively. This gives us only slightly modified gradients $G_{m}(U),G_{m}(B)$ and Hessians $H_{m}(U),H_{m}(B)$ with the subscript ${\;}_{m}$ denoting the multimodal model. This does not complicate the computation and the same parameter and hyperparameter updates hold as detailed in Section 2.3.2 by simply replacing the Hessian with the multimodal variants. The final algorithm in pseudo-code is given in Algorithm 1.

#### 2.4.3 Hyperparameters and computational considerations

The overall algorithm can be entirely hyperparameter-free as step sizes γ below 1 lead to convergence using ALS within tens to hundreds of steps and ARD can shrink unnecessary latent variables. In practice, we use γ=0.1 and k=10 latents in our experiments. Alternatively, the computed marginal likelihood can be used to select the number of latent variables by running the algorithm for different numbers of latents *k* as in Bayesian PCA (Bishop 1998). The algorithm scales cubically in the Hessian sizes, which is $O(k^{3})$ for the Hessian of B due to the Kronecker-factored structure and $O(p^{3}k^{3})$ for the Hessian of U. The latter can be expensive when the number of latents and pathways is large, in which case a diagonal approximation is still viable and implemented in our code.

## 3 Results

We developed and implemented a novel model for factor analysis, PathFA that leverages pathway information for multimodal data integration and incorporates a probabilistic framework with automatic hyperparameter optimization. It bridges the gap between two existing approaches, MOFA and PLIER. PathFA enables pathway-based interpretation of multimodal factor analysis, specifically suited for small cohorts.

In the sections that follow, we demonstrate how the inclusion of prior information enhances the reconstruction log-likelihood. We observe improvements in the reconstruction of each individual modality and in the convergence behavior when a second data modality is incorporated. Additionally, we showcase the practical utility of our approach through its application to a subset of patient samples from the Swiss Tumor Profiler cohort in a real-world setting.

### 3.1 Including prior information improves reconstruction

On the synthetic data generated using MSigDB Hallmark as described in Section 2.1.3, we observe that PathFA has clear advantages over PLIER and MOFA in terms of reconstruction performance, sample efficiency, and inferring observation noises on the markers of both proteomics and transcriptomics markers. We use the public implementations and propose default parameters of PLIER and MOFA and set the number of latent variables for all methods to the ground truth 8.

With access to the ground truth parameters, we can measure the reconstruction performance on test data and fitting of heteroscedastic marker-level observation noises. That is, we use the method in question on a set of *m* samples to infer the model and then fix the model before inferring loadings for a held-out set of $m_{\text{test}}$ samples, $Y_{r}^{\left(\text{test}\right)}$ and $Y_{p}^{\left(\text{test}\right)}$. With inferred loadings, we reconstruct the test data and obtain the estimates ${\hat{Y}}_{r}^{\left(\text{test}\right)}$ and ${\hat{Y}}_{p}^{\left(\text{test}\right)}$. The reconstruction log-likelihood,

(12) $$\text{log}\;p({\hat{Y}}_{p}^{\left(\text{test}\right)}|C,U,B,\sigma )=\text{log}\;N({\hat{Y}}_{p}^{\left(\text{test}\right)};CUB,\sigma I),$$

denotes an entry-wise Gaussian distribution on the matrices and holds for either proteomics or transcriptomics observation and reconstruction. This allows to assess how well the fitted model can infer representations from noisy observations. This is only possible to measure in a simulation. We have an upper bound on the performance with the ground truth reconstruction ${\hat{Y}}_{x}^{\left(\text{test}\right)}=C_{x}UB$ for any data modality *x*. With these parameters, we can reconstruct the noise-free observation, $\hat{Y_{r}}$ and $\hat{Y_{p}}$, and assess this reconstruction under the true model in terms of log-likelihood. Further, we assess how well our probabilistic model can recover the true observation noise of the model in comparison to MOFA, which does not use prior knowledge C.

**Figure 2.**
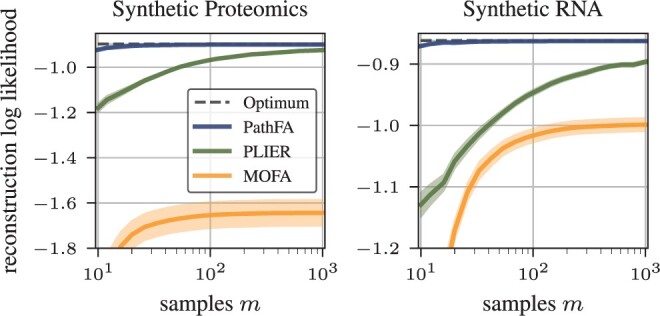
Reconstruction log-likelihood on the synthetic benchmark data as a function of samples for proteomics and RNA averaged over 20 runs. Shaded regions denote twice the standard error about the mean. Unimodal PathFA, unimodal PLIER, and MOFA are compared to optimal attainable performance (dotted line).

**Figure 3.**
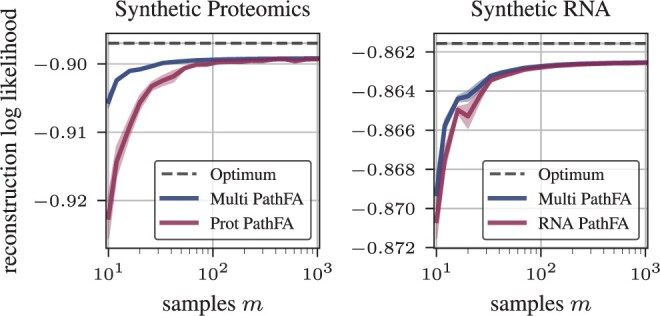
Reconstruction log-likelihood of multimodal PathFA in comparison to the unimodal variant as depicted in Fig. 2.

**Figure 4.**
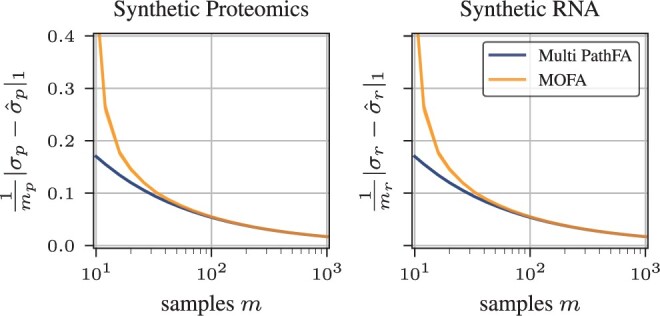
Average absolute error of marker-level observation noises on synthetic benchmark of Multi-PathFA and MOFA for both proteomics and RNA. The lines show the average over 20 runs and shaded regions two standard errors.

We compare the reconstruction log-likelihood across different models using one modality, synthetic proteomics, or synthetic RNA, in Fig. 2. The trends of the reconstruction log-likelihood across different sample sizes are consistent between both modalities. MOFA without pathway information shows the lowest reconstruction log-likelihood. PathFA further improves over PLIER, especially for small sample sizes, due to automatic hyperparameter optimization and focus on pathway-level factor analysis. Figure 3 further shows that using both omics improves reconstruction of them individually, especially for proteomics, which appears to provide less evidence for the loadings. Already around m=30 samples suffice for the loadings to converge with PathFA. Like MOFA, PathFA can infer marker-level heteroscedastic observation noises and converges faster in terms of the samples needed due to the prior information on pathways, as apparent in Fig. 4.

### 3.2 PathFA performs well in small-sample cohorts

For the purpose of investigating how multimodality contribute towards the performance of PathFA, we compare the change in reconstruction likelihood over sample number between PathFA in a unimodal setting with synthetic trancscriptomics or proteomics data only (blue) versus PathFA with multimodal data (purple) in Fig. 3. We then use the unimodal or multimodal model, to evaluate the reconstruction log-likelihood and the observation noise, respectively, on transcriptomics and proteomics data separately.

Including both modalities strictly improves the reconstruction performance, especially for small sample sizes, which are common in realistic biomedical datasets, as also in our case in Section 3.3.

To show the effect of prior information, we also compare PathFA against multi-omics factor-analysis (MOFA). Unlike MOFA, we integrate modalities on a pathway level. This provides not only prior information but also enables a pathway-based multimodal factor analysis that should simplify the interpretation of the results. Generally, PathFA demonstrates similar behaviour to MOFA with respect to fitting observation noise. However, in the range of small sample numbers, the utility of prior information takes effect leading to much smaller observation noise error in PathFA.

### 3.3 Comparing pathway loadings to cell type composition

To demonstrate the utility of our approach, we also benchmark PathFA on a subset of patient samples from the TuPro cohort. This cohort is well suited for a benchmark study as it provides paired bulk proteomics and transcriptomics measurements for each sample. In addition, CyTOF data, also generated for all samples, provides single-cell measurements of several protein markers that we able to use as an independent estimate of the ground-truth cell-type composition. We use a subset of 42 samples from ovarian cancer patients as well as 34 samples from the melanoma cancer patients that have available CyTOF compositions, clinical information of the melanoma cohort, and tumor heterogeneity scores of the ovarian cancer cohort.

**Figure 5.**
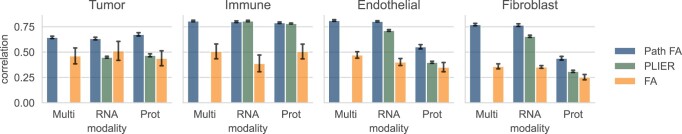
This figure shows the Pearson correlation coefficients of pathway loadings with cell-type content (based on ground truth computed from CyTOF data) across 42 ovarian cancer samples for the four most common cell-types. MSigDB cell-type pathways were used for PathFA across all experiments. Multirefers to the multimodal setting, while RNA and Prot refers towards results based on transcriptomic and proteomic data only. FA refers to factor analysis respectively MOFA in the multisetting. For both, correlation with latent factors is computed. PLIER can only be used in unimodal setting.

Here, we train PathFA using the MSigDB Hallmark pathways and evaluate the quality of learned representations by their ability to correlate with cell-type composition. Figure 5 shows the highest correlating pathway loadings from PathFA and baseline approaches with CyTOF estimates of cell-type content in a unimodal and multimodal setting. In the unimodal and multimodal setting, PathFA consistently outperforms standard factor analysis (respectively, MOFA in the multimodal setting), for which we use standard instead of pathway loadings, as well as PLIER with respect to deriving factors that are highly correlated with cell-type content. These results are similar with the corresponding analysis in the melanoma cancer cohort (see supplemental Fig. S1), however, with systematically lower correlation in endothelial and fibroblasts across all approaches. The gain in the multimodal setting over the unimodal approach is small with respect to this evaluation. While the multimodal approach does improve over a proteomic-only approach, the performance over an RNA-only approach is similar. The difference in marker resolution between transcriptomics and proteomics data, may explain this trend.

Next, we identify pathways associated with cell-type composition by computing correlations between the CyTOF estimate and pathway loadings. Thus, we run PathFA using cell-type marking pathways and report the top five highest correlated pathways to each cell type in supplemental Fig. S3 in the Melanoma case. These pathways seem indicative of their respective cell types, melanoma pathway for tumor cell type, T cell pathways for immune cell type, Cancer associated fibroblasts (CAFs) pathways for fibroblast cell type, and endothelial melanoma pathways for endothelial cell types. We observe similar results in the Ovarian cancer cohort (see supplemental Fig. S2).

We further test the efficacy of PathFA when the pathway masks are perturbed and thus the prior information might not be fully accurate. In supplemental Fig. S6, we find that the average cell-type correlation over all four types remains high even for a substanial amount of flipped pathway-marker associations. The same figure shows that on the synthetic data, this is not necessarily the case and the reconstruction log likelihood suffers from flipping associations.

### 3.4 Associating pathway loadings to patient survival in a melanoma cohort

**Figure 6.**
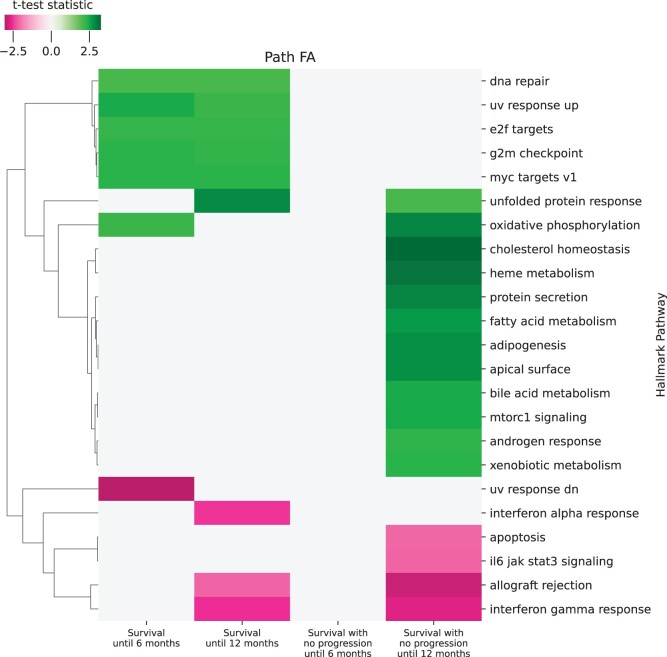
Significant associations between survival and progression in relationship to pathway loadings from PathFA. The color refers to the normalized mean difference between the two patient groups in each column, for significant associations only. The clustering tree implies the presence of three clusters, two of which seem to be higher expressed [top (5 pathways) and middle (12 pathways)], and the remaining one appears to be down-regulated in reference to the according patient group (*y*-axis).

Conceptually, PathFA is designed to enable easier interpretation of the results of a multimodal factor analysis on the level of pathways. To investigate this further, we look at survival data as well as time to progression of the melanoma patient cohort. We analyzed the association of the pathway loadings of PathFA in the multimodal setting (transcriptomics and proteomics) with survival and patient progression. Each patient is given a binary label for four categories, if the patient has survived for at least 6 and 12 months, and if the patient has survive without progression for 6 and 12 months. Then, a *t*-test is performed between each label and the pathway loadings. A clustermap over the *t*-test statistics for associated pathway loadings are shown in Fig. 6

We observe three major clusters in the melanoma gene set analysis. Cluster 1, top 5 pathways, consists out of upgregulated gene sets associated with proliferation and DNA damage, while cluster 2, middle 12 pathways, encompasses various process categories, such as pathways, metabolism, and development. The remaining cluster 3, bottom 6 pathways, shows enrichment in downregulated gene sets, primarily related to immune responses, DNA damage, and pathways. Furthermore, in line with current literature reporting downregulated gene sets associated with worse clinical outcomes (Garg *et al.* 2021), hallmark gene sets like hallmark_allograft_rejection and hallmark_interferon_gamma_response are enriched in the group of patients with poor outcomes, including those who either succumbed to the disease or progressed within 12 months (see Fig. 6). When compared to a single-modal setting using transcriptomics and proteomics data separately, it becomes apparent that most of the observed signal originates from the transcriptomics data, with two pathways being uniquely identified through proteomics signal (see supplemental Fig. S4).

### 3.5 Pathway loadings are associated with tumor heterogeneity in ovarian cancer

**Figure 7.**
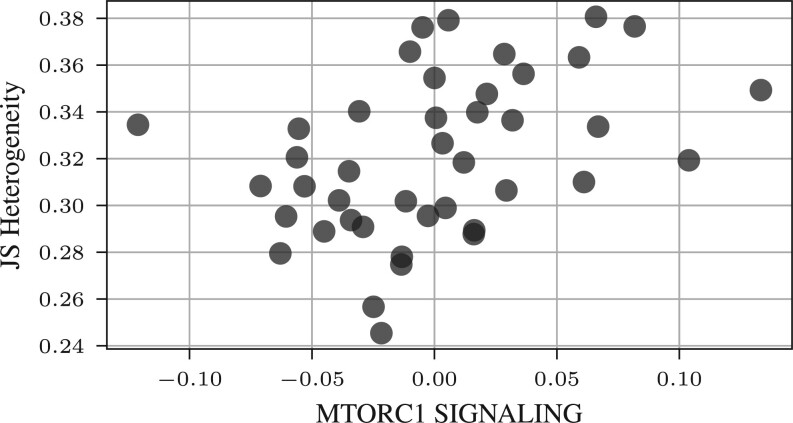
Scatter plot of Jensen–Shannon tumor heterogeneity computed on CyTOF measurements of tumor cells (*y*-axis) and mTORC1 pathway loading generated by PathFA. Each dot represents a ovarian cancer patient sample. The Pearson’s correlation is 0.41 (*P*-value = .0075).

We proceed to evaluate the performance of PathFA in the ovarian cancer cohort, focusing on the challenge of tumor heterogeneity (Vázquez-García *et al.* 2022). The tumor heterogeneity is computed based on the Jenson-Shannon divergence from CyTOF data on tumor cells. Similar to the previous tasks, PathFA achieves the highest correlation between latent factors and tumor heterogeneity (see supplemental Fig. S5). When comparing heterogeneity estimates derived from tumor cell populations using CyTOF, we observe significant correlations. Notably, OXIDATIVE_PHOSPHORYLATION and UNFOLDED_PROTEIN_RESPONSE shows positive correlations (see Table 1) including mTORC1 (see Fig. 7). This suggests associations between endoplasmic reticulum stress, metabolic processes and tumor heterogeneity in ovarian cancer and is consistent with previous reports on pro-survival mechanisms (Madden *et al.* 2019) and metabolic processes (Sancho *et al.* 2016).

**Table 1.** This is a list of 10 MSigDB Hallmark pathways that have the highest Pearson correlation with tumor heterogeneity score on 42 ovarian cancer samples.

Rank	MSigDB Hallmark pathway	Correlation	*P*-value
1	MTORC1_SIGNALING	0.41	.01
2	GLYCOLYSIS	0.34	.03
3	G2M_CHECKPOINT	0.32	.04
4	E2F_TARGETS	0.31	.04
5	MYC_TARGETS_V1	0.29	.06
6	OXIDATIVE_PHOSPHORYLATION	0.29	.06
7	UNFOLDED_PROTEIN_RESPONSE	0.24	.12
8	DNA_REPAIR	0.23	.14
9	MYC_TARGETS_V2	0.23	.15
10	PEROXISOME	0.22	.16

## 4 Discussion and conclusion

In this study, we introduced PathFA, a novel multimodal factor analysis approach tailored for genomic data, employing Bayesian hyperparameter optimization to seamlessly integrate transcriptomic and proteomic data using pathway sets. This method innovatively addresses heteroscedasticity in markers and modalities, a prevalent challenge in experimental measurements. Through various experiments, we established that incorporating known prior information not only enhances reconstruction quality but also improves data efficiency through Bayesian hyperparameter updates. Our probabilistic formulation, especially the automatic optimization of hyperparameters, marks an improvement over PLIER, which lacks this adaptability.

A notable advancement of PathFA is its ability to provide immediate interpretability of pathway activities, in contrast to traditional approaches that often rely on post-hoc gene set enrichment analysis. This immediate interpretability is exemplified in our analysis of a cancer patient cohort from the Tumor Profiler Project, where PathFA successfully correlates pathway activities with crucial biological aspects such as cell-type composition, survival, and tumor heterogeneity. These capabilities illustrate the potential of PathFA in elucidating complex biological relationships and hypothesis generation.

PathFA is implemented using a Gaussian likelihood. This provides a robust foundation for different data modalities. In the future, it may be desirable to expand this framework to allow other likelihood functions such as the negative binomial, often used in context of read count data. PathFA may also expand across other biological data-types for which groupings (e.g. pathways masks) of individual markers (e.g. genes) is possible across the modalities. Furthermore, our approach extends beyond the typical limitations of analyzing dual modalities. It is readily adaptable to include additional data types, promising broader applicability as genomic profiling technologies evolve. A potential complication of such multimodal datasets lie in the differences in cellular composition between parallel tissues slices. Looking forward, we envision enhancements to PathFA that incorporate modality-specific prior information that are in addition flexible to these tissues effects, further augmenting its analytical power.

In summary, PathFA represents a significant step forward in multimodal genomic analysis, offering robustness, flexibility, and direct interpretability. Its development aligns with the ongoing advancements in profiling technologies, positioning it as a valuable tool in understanding complex biological systems and disease mechanisms.

## Supplementary Material

btae216_Supplementary_Data

## Data Availability

Data will be made available with the upcoming Tumor Profiler Marker Publications. Details will be made available under http://tu-pro.ch/download/pathFA/.
