# Supplementary material for: Probabilistic pathway-based multimodal factor analysis
Source: Bioinformatics. 2024 Jun 28;40(Suppl 1):i189–98. doi: 10.1093/bioinformatics/btae216 (PMC11256960; doi:10.1093/bioinformatics/btae216)
Supplement: btae216_Supplementary_Data [file btae216_supplementary_data.pdf]

## 5 Supplement

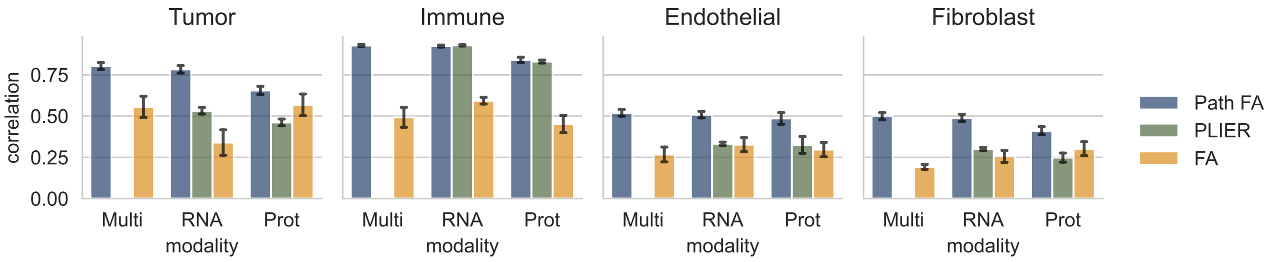


Fig. S1: Correlation of pathway loadings with cell-type content (ground truth based on CyTOF data) across 34 melanoma samples for the four most common cell-types. MSigDB c8 cell type pathways. The y-axis shows the Pearson correlation coefficient. Multi refers to the multimodal setting.

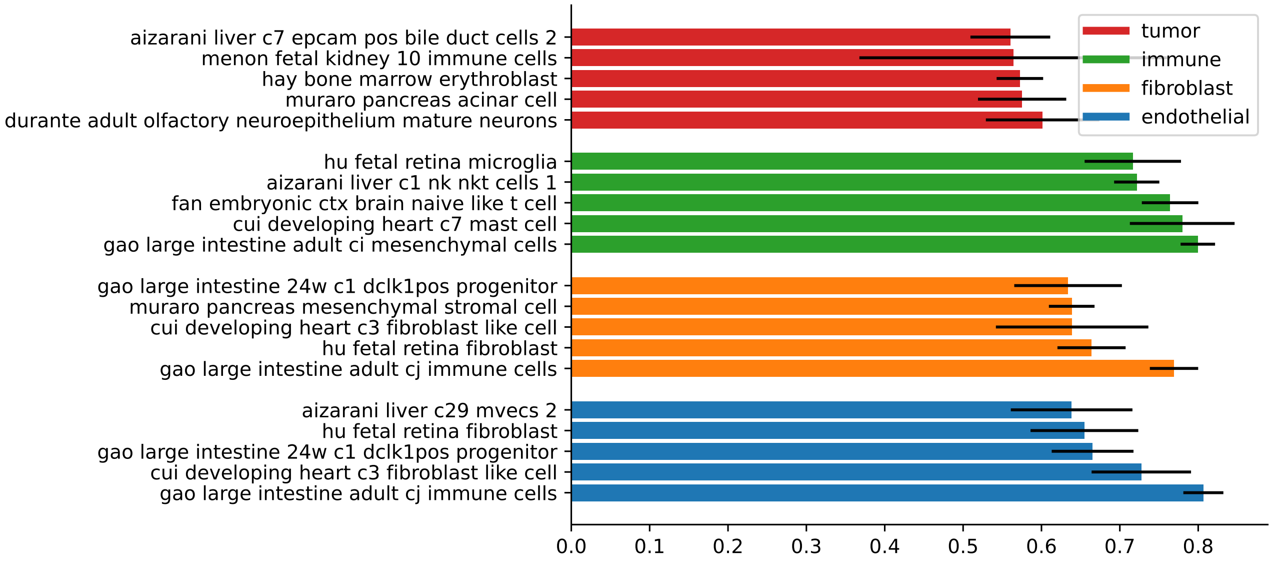


Fig. S2: Pearson correlation of the top 5 most correlated (pathway) loadings to each cell type in ovarian cancer samples. MSigDB C8 cell type genesets are used as pathways.

| Rank | MSigDB Hallmark Pathway | Correlation | p-value |
|---|---|---|---|
| 1 | MTORC1_SIGNALING | 0.47 | 0.01 |
| 2 | GLYCOLYSIS | 0.35 | 0.02 |
| 3 | OXIDATIVE_PHOSPHORYLATION | 0.33 | 0.03 |
| 4 | G2M_CHECKPOINT | 0.31 | 0.04 |
| 5 | PEROXISOME | 0.26 | 0.10 |
| 6 | E2F_TARGETS | 0.25 | 0.10 |
| 7 | UNFOLDED_PROTEIN_RESPONSE | 0.25 | 0.12 |
| 8 | MYC_TARGETS_V1 | 0.23 | 0.14 |
| 9 | MYC_TARGETS_V2 | 0.22 | 0.17 |
| 10 | SPERMATOGENESIS | 0.19 | 0.23 |

Table S1. This table corresponds to Table 1 but reports the Spearman correlation instead of Pearson correlation.

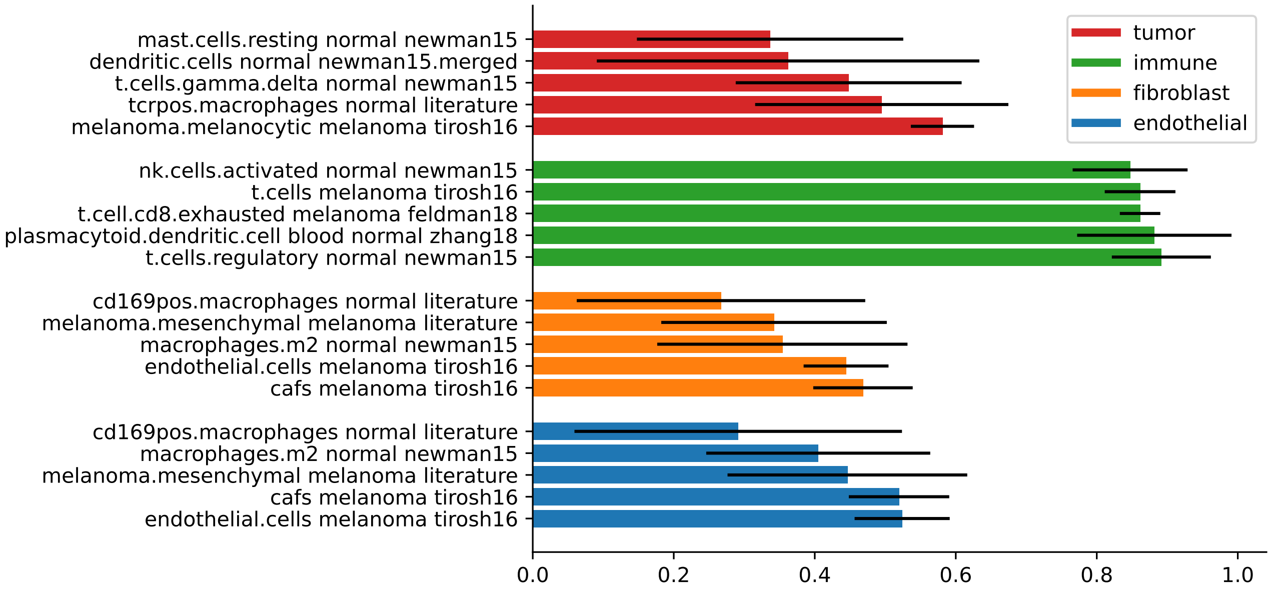


Fig. S3: Pearson correlation of the top 5 most correlated (pathway) loadings to each cell type in Melanoma samples. The pathways used are curated by the Tumor Profiler Melanoma node and chosen for cell-type composition.

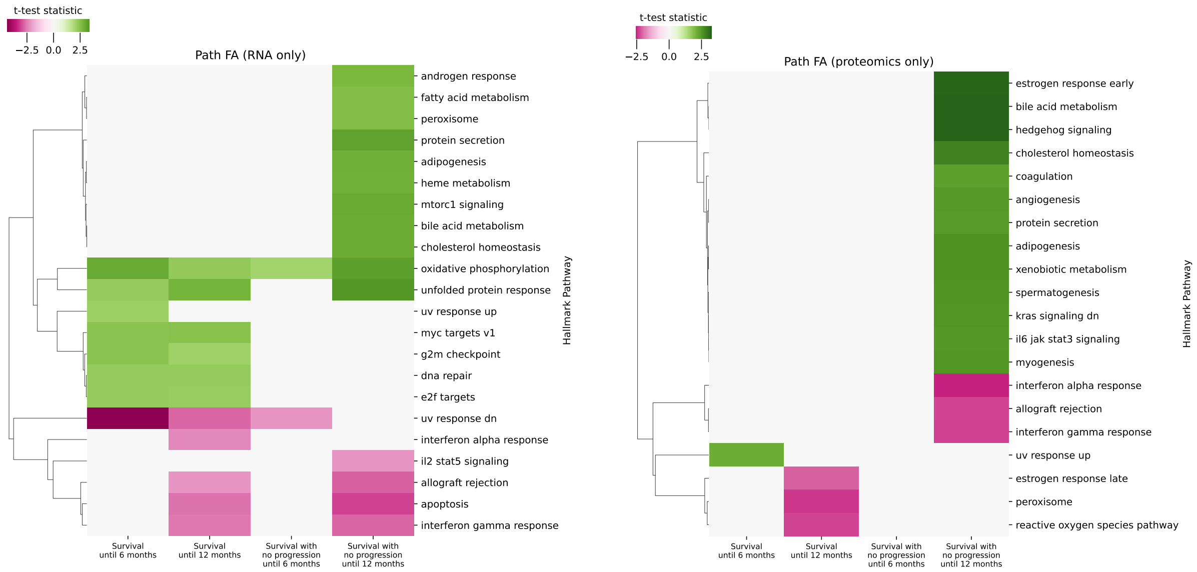


(a) RNA-only loadings

(b) proteomics-only loadings

Fig. S4: Significant associations between survival and progression in relationship to *unimodal* pathway loadings from Path FA. The color refers to the normalized mean difference between the groups for significant associations only.

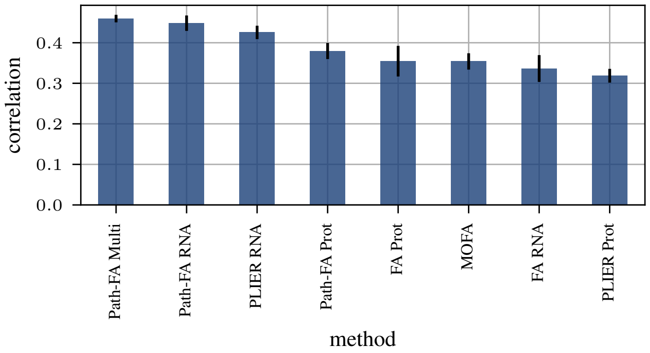


Fig. S5: Pearson correlation of (pathway) loadings with tumor heterogeneity in ovarian samples.

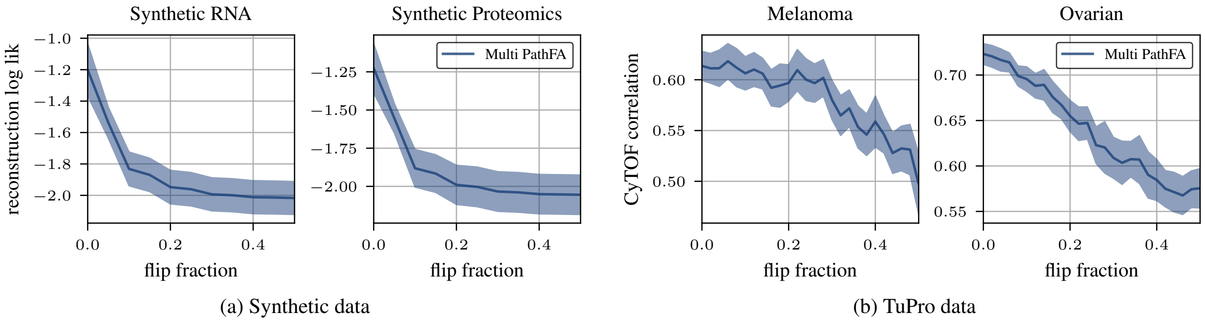


Fig. S6: Performance on synthetic (a) and real-world TuPro (b) data when randomly flipping a fraction of bits of every geneset in the masks. The performance degrades, as expected, but on real data leads to less performance deterioration in terms of average cell-type correlation. The TuPro example uses Hallmark genesets.

## 5.1 Tumor Profiler authors

Rudolf Aebersold[5], Melike Ak[33], Faisal S Al-Quaddoomi[12,22], Silvana I Albert[10], Jonas Albinus[10], Ilaria Alborelli[29], Sonali Andani[9,22,31,36], Per-Olof Attinger[14], Marina Bacac[21], Daniel Baumhoer[29], Beatrice Beck-Schimmer[44], Niko Beerenwinkel[7,22], Christian Beisel[7], Lara Bernasconi[32], Anne Bertolini[12,22], Bernd Bodenmiller[11,40], Ximena Bonilla[9], Lars Bosshard[12,22], Byron Calgua[29], Ruben Casanova[40], Stéphane Chevrier[40], Natalia Chicherova[12,22], Ricardo Coelho[23], Maya D'Costa[13], Esther Danenberg[42], Natalie R Davidson[9], Monica-Andreea Dragan[7], Reinhard Dummer[33], Stefanie Engler[40], Martin Erkens[19], Katja Eschbach[7], Cinzia Esposito[42], André Fedier[23], Pedro F Ferreira[7], Joanna Ficek-Pascual[1,9,16,22,31], Anja L Frei[36], Bruno Frey[18], Sandra Goetze[10], Linda Grob[12,22], Gabriele Gut[42], Detlef Günther[8], Pirmin Haeuptle[3], Viola Heinzelmann-Schwarz[23,28], Sylvia Herter[21], Rene Holtackers[42], Tamara Huesser[21], Alexander Immer[9,17], Anja Irmisch[33], Francis Jacob[23], Andrea Jacobs[40], Tim M Jaeger[14], Katharina Jahn[7], Alva R James[9,22,31], Philip M Jermann[29], André Kahles[9,22,31], Abdullah Kahraman[22,36], Viktor H Koelzer[36,41], Werner Kuebler[30], Jack Kuipers[7,22], Christian P Kunze[27], Christian Kurzeder[26], Kjong-Van Lehmann[2,4,9,15], Mitchell Levesque[33], Ulrike Lischetti[23], Flavio C Lombardo[23], Sebastian Lugert[13], Gerd Maass[18], Markus G Manz[35], Philipp Markolin[9], Martin Mehnert[10], Julien Mena[5], Julian M Metzler[34], Nicola Miglino[35,41], Emanuela S Milani[10], Holger Moch[36], Simone Muenst[29], Riccardo Murri[43], Charlotte KY Ng[29,39], Stefan Nicolet[29], Marta Nowak[36], Monica Nunez Lopez[23], Patrick GA Pedrioli[6], Lucas Pelkmans[42], Salvatore Piscuoglio[23,29], Michael Prummer[12,22], Prélot, Laurie[9,22,31], Natalie Rimmer[23], Mathilde Ritter[23], Christian Rommel[19], Maria L Rosano-Gonzalez[12,22], Gunnar Rätsch[1,6,9,22,31], Natascha Santacroce[7], Jacobo Sarabia del Castillo[42], Ramona Schlenker[20], Petra C Schwalie[19], Severin Schwan[14], Tobias Schär[7], Gabriela Senti[32], Wenguang Shao[10], Franziska Singer[12,22], Sujana Sivapatham[40], Berend Snijder[5,22], Bettina Sobottka[36], Vipin T Sreedharan[12,22], Stefan G Stark[9,22,31], Daniel J Stekhoven[12,22], Tanmay Tanna[7,9], Alexandre PA Theocharides[35], Tinu M Thomas[9,22,31], Markus Tolnay[29], Vinko Tosevski[21], Nora C Toussaint[12,22], Mustafa A Tuncel[7,22], Marina Tusup[33], Audrey Van Drogen[10], Marcus Vetter[25], Tatjana Vlajnic[29], Sandra Weber[32], Walter P Weber[24], Rebekka Wegmann[5], Michael Weller[38], Fabian Wendt[10], Norbert Wey[36], Andreas Wicki[35,41], Mattheus HE Wildschut[5,35], Bernd Wollscheid[10], Shuqing Yu[12,22], Johanna Ziegler[33], Marc Zimmermann[9], Martin Zoche[36], Gregor Zuend[37]

[1]AI Center at ETH Zurich, Andreasstrasse 5, 8092 Zurich, Switzerland, [2]Cancer Research Center Cologne-Essen, University Hospital Cologne, Cologne, Germany, [3]Cantonal Hospital Baselland, Medical University Clinic, Rheinstrasse 26, 4410 Liestal, Switzerland, [4]Center for Integrated Oncology Aachen (CIO-A), Aachen, Germany, [5]ETH Zurich, Department of Biology, Institute of Molecular Systems Biology, Otto-Stern-Weg 3, 8093 Zurich, Switzerland, [6]ETH Zurich, Department of Biology, Wolfgang-Pauli-Strasse 27, 8093 Zurich, Switzerland, [7]ETH Zurich, Department of Biosystems Science and Engineering, Mattenstrasse 26, 4058 Basel, Switzerland, [8]ETH Zurich, Department of Chemistry and Applied Biosciences, Vladimir-Prelog-Weg 1-5/10, 8093 Zurich, Switzerland, [9]ETH Zurich, Department of Computer Science, Institute of Machine Learning, Universitatstrasse 6, 8092 Zurich, Switzerland, [10]ETH Zurich, Department of Health Sciences and Technology, Otto-Stern-Weg 3, 8093 Zurich, Switzerland, [11]ETH Zurich, Institute of Molecular Health Sciences, Otto-Stern-Weg 7, 8093 Zurich, Switzerland, [12]ETH Zurich, NEXUS Personalized Health Technologies, Wagistrasse 18, 8952 Zurich, Switzerland, [13]F. Hoffmann-La Roche Ltd, Grenzacherstrasse 124, 4070 Basel, Switzerland, [14]F. Hoffmann-La Roche Ltd, Grenzacherstrasse 124, 4070 Basel, Switzerland, , [15]Joint Research Center Computational Biomedicine, University Hospital RWTH Aachen, Aachen, Germany, [16]Life Science Zurich Graduate School, Biomedicine PhD Program, Winterthurerstrasse 190, 8057 Zurich, Switzerland, [17]Max Planck ETH Center for Learning Systems, , [18]Roche Diagnostics GmbH, Nonnenwald 2, 82377 Penzberg, Germany, [19]Roche Pharmaceutical Research and Early Development, Roche Innovation Center Basel, Grenzacherstrasse 124, 4070 Basel, Switzerland, [20]Roche Pharmaceutical Research and Early Development, Roche Innovation Center Munich, Roche Diagnostics GmbH, Nonnenwald 2, 82377 Penzberg, Germany, [21]Roche Pharmaceutical Research and Early Development, Roche Innovation Center Zurich, Wagistrasse 10, 8952 Schlieren, Switzerland, [22]SIB Swiss Institute of Bioinformatics, Lausanne, Switzerland, [23]University Hospital Basel and University of Basel, Department of Biomedicine, Hebelstrasse 20, 4031 Basel, Switzerland, [24]University Hospital Basel and University of Basel, Department of Surgery, Brustzentrum, Spitalstrasse 21, 4031 Basel, Switzerland, [25]University Hospital Basel, Brustzentrum & Tumorzentrum, Petersgraben 4, 4031 Basel, Switzerland, [26]University Hospital Basel, Brustzentrum, Spitalstrasse 21, 4031 Basel, Switzerland, [27]University Hospital Basel, Department of Information- and Communication Technology, Spitalstrasse 26, 4031 Basel, Switzerland, [28]University Hospital Basel, Gynecological Cancer Center, Spitalstrasse 21, 4031 Basel, Switzerland, [29]University Hospital Basel, Institute of Medical Genetics and Pathology, Schönbeinstrasse 40, 4031 Basel, Switzerland, [30]University Hospital Basel, Spitalstrasse 21/Petersgraben 4, 4031 Basel, Switzerland, [31]University Hospital Zurich, Biomedical Informatics, Schmelzbergstrasse 26, 8006 Zurich, Switzerland, [32]University Hospital Zurich, Clinical Trials Center, Ramistrasse 100, 8091 Zurich, Switzerland, [33]University Hospital Zurich, Department of Dermatology, Gloriastrasse 31, 8091 Zurich, Switzerland, [34]University Hospital Zurich, Department of Gynecology, Frauenklinikstrasse 10, 8091 Zurich, Switzerland, [35]University Hospital Zurich, Department of Medical Oncology and Hematology, Ramistrasse 100, 8091 Zurich, Switzerland, [36]University Hospital Zurich, Department of Pathology and Molecular Pathology, Schmelzbergstrasse 12, 8091 Zurich, Switzerland, [37]University Hospital Zurich, Ramistrasse 100, 8091 Zurich, Switzerland, [38]University Hospital and University of Zurich, Department of Neurology, Frauenklinikstrasse 26, 8091 Zurich, Switzerland, [39]University of Bern, Department of BioMedical Research, Murtenstrasse 35, 3008 Bern, Switzerland, [40]University of Zurich, Department of Quantitative Biomedicine, Winterthurerstrasse 190, 8057 Zurich, Switzerland, [41]University of Zurich, Faculty of Medicine, Zurich, Switzerland, [42]University of Zurich, Institute of Molecular Life Sciences, Winterthurerstrasse 190, 8057 Zurich, Switzerland, [43]University of Zurich, Services and Support for Science IT, Winterthurerstrasse 190, 8057 Zurich, Switzerland, [44]University of Zurich, VP Medicine, Künstlergasse 15, 8001 Zurich, Switzerland
